# Intracellular calcium current disorder and disease phenotype in *OBSCN* mutant iPSC-based cardiomyocytes in arrhythmogenic right ventricular cardiomyopathy

**DOI:** 10.7150/thno.45172

**Published:** 2020-09-14

**Authors:** Peipei Chen, Ying Xiao, Yuanpin Wang, Zhifa Zheng, Lianfeng Chen, Xufei Yang, Jingyi Li, Wei Wu, Shuyang Zhang

**Affiliations:** 1 Department of Cardiology, Peking Union Medical College Hospital, Chinese Academy of Medical Sciences and Peking Union Medical College, Beijing 100730, China.; 2 Central Research Laboratory, Peking Union Medical College Hospital, Chinese Academy of Medical Sciences and Peking Union Medical College, Beijing, China.

**Keywords:** arrhythmogenic right ventricular cardiomyopathy, induced pluripotent stem cells-based cardiomyocytes, obscurin

## Abstract

Obscurin participates in the development of striated muscles and maintenance of the functional sarcoplasmic reticulum. However, the role of obscurin in arrhythmogenic right ventricular cardiomyopathy (ARVC) is not well understood. We aimed to study the novel obscurin mutations in the pathogenesis of ARVC and the underlying mechanisms.

**Methods:** We generated induced pluripotent stem cells (iPSC) through retroviral reprogramming of peripheral blood mononuclear cells isolated from a 46-year-old female diagnosed with ARVC, carrying a mutation in *OBSCN*. The cells differentiated into functional iPSC-based cardiomyocytes (iPSC-CMs), whose phenotype was determined by transmission electron microscopy, electrophysiological description, immunofluorescence staining, and Oil Red O staining. Molecular characterization was performed by bioinformatic analyses, and identification by quantitative real-time polymerase chain reaction (qRT-PCR) and Western blotting.

**Results:** ARVC-iPSC-CMs mutation in *OBSCN* showed significant accumulation of lipids, increased pleomorphism, irregular Z-bands, and increased L type calcium currents. Functional enrichment analysis identified pathways involved in focal adhesion and structure formation; the adipocytokines and PPAR signaling pathways were also activated in the ARVC group. Moreover, our results from ultra-high-resolution microscopy, qRT-PCR and Western blotting confirmed that the mutant OBSCN protein and its anchor protein, Ank1.5, showed structural disorder and decreased expression, but there was increased expression of junctional protein N-Cadherin. Further analysis revealed the gene expression of other desmosomal proteins in ARVC-iPSC-CMs was also decreased but some adipogenesis pathway-related proteins (PPARγ, C/EBPα, and FABP4) were increased.

**Conclusion:** A novel frameshift mutation in *OBSCN* caused phenotypic alteration accompanied by disrupted localization and decreased expression of its anchoring protein Ank1.5. Furthermore, there was an accumulation of lipids with an increase in fatty fibrosis area and myocardial structural disorder, possibly leading to dysrhythmia in calcium channel-related myocardial contraction. These observations suggested the possibility of attenuating ARVC progression by therapeutic modulation of OBSCN expression.

## Introduction

Arrhythmogenic right ventricular cardiomyopathy (ARVC) is regarded as a rare type of cardiomyopathy, featuring progressive fibrofatty and adipose tissue replacement of the right ventricle and life-threatening ventricular arrhythmias 1. Etiology of this condition was reported to be associated with gene mutations, with around fifty percent of cases harboring mutations in genes encoding for desmosomal proteins, such as desmoglein 2 (DSG2), desmoplakin, plakoglobin, and plakophilin 2 (PKP2), as well as desmocollin 2 2. Patients with symptomatic ARVC have difficulty for a preliminary biopsy of cardiac tissue because of the increased cardiac perforation risk. Animal models have provided insights into the pathogenesis of ARVC. However, due to the distinctions in the electrophysiological functions between the hearts of animals and humans, the elucidation of the involved mechanisms and the applicability of experimental data are restricted. These limitations have hampered the exploration of potential therapies for the management of human ARVC. Several research groups have successfully modeled induced pluripotent stem cell-type cardiomyocytes (iPSC-CMs) from patients with many hereditary cardiac ion channel diseases that were different from the well-known subtypes of the long-QT and Brugada syndrome in recent years 3-6. The current study aimed to establish a model of iPSC-CMs for patients with ARVC, elucidate the relationship between novel mutations in the *OBSCN* gene and ARVC disease phenotypes, and explore the underlying mechanisms of pathogenesis.

Obscurin is known to exhibit a close association with large proteins, and can interact with complexes of titin, myomesin, and small ankyrin-1 as is evident in striated muscles. It is considered a structural protein that connects the sarcomere M-line with the sarcoplasmic reticulum 7, 8. The *OBSCN* gene is mainly expressed in myocytes and its protein product, obscurin is distributed within sarcomeres, and is known to participate in the development of striated muscles and maintaining the functional sarcoplasmic reticulum. Mutations in the *OBSCN* gene have been reported to be associated with heart diseases, such as hypertrophic cardiomyopathy and left ventricular noncompaction. There are no reports suggesting that mutations in the *OBSCN* gene cause ARVC. However, evidence indicates that variations in the obscurin domain might cause deregulation of calcium homeostasis and arrhythmias. Thus, it is important to investigate whether mutations in the *OBSCN* gene could cause ARVC, identify its underlying pathogenesis, understand obscurin's function, and find potential therapeutic targets for the management of ARVC.

## Results

### Gene sequencing data

**Figure 1:**
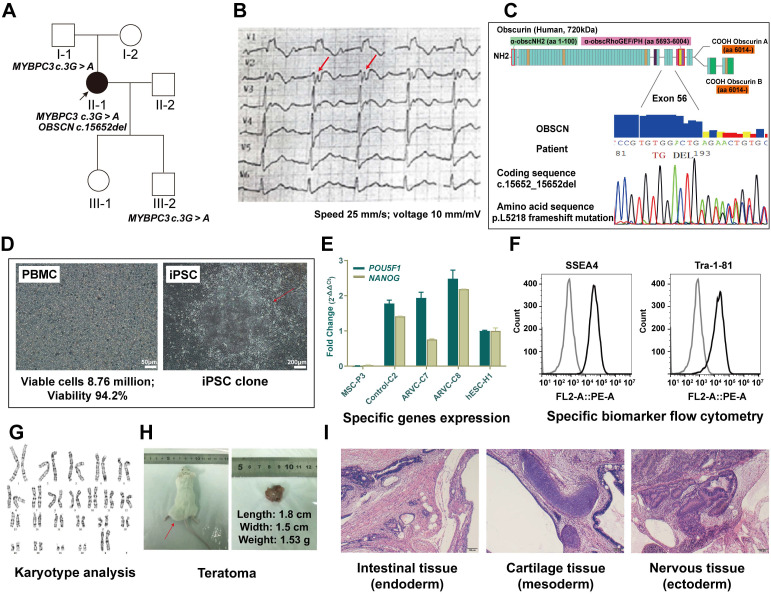
** Information on the ARVC patient and establishment of induced pluripotent stem cell**. (A) Squares indicate males; circles, females; filled-in symbols, clinically affected individuals; blank symbols, clinically unaffected. (B)Twelve-lead electrocardiography of patient with ARVC. T-wave inversion in leads V1 to V3, and epsilon (red arrow) wave appears after the QRS complex. (C) Schematic diagram representing the deletion mutation in obscurin (OBSCN), resulting in frameshift mutation at position 5218. (D) Morphology of peripheral blood mononuclear cells (PBMC) from a patient and establishment of ARVC-iPSCs on number 1 passage, clone 1. (E) iPSC lines express similar RNA levels of the *POU5F1* and *NODAL* pluripotency-related genes as those observed in hESCs, as revealed by qRT-PCR analysis. (F) iPSCs are stained with pluripotency markers (SSEA-4 and TRA-1-60) as indicated by flow cytometry. (G) iPSCs retained long telomeres and a normal karyotype. (H, i) iPSCs contributed to typical teratomas, made up of distinct structures representing the 3-germ layers (neural rosettes and retinal pigmented epithelium [ectoderm], intestinal epithelium [endoderm] and cartilage, bone and fatness, as well as muscle [mesoderm]).

We sequenced the whole exomes of the patient's family members in this study. Proband (II-1) carried *OBSCN* gene at c.15652_15652del (p. Leu5218fs). Proband, proband's father (I-1) and son (III-2) harbored c.3G > A (p. Met1Ile) mutation, whereas the proband's mother (I-2), husband, and daughter carried no mutations in *OBSCN* or *MYBPC3* genes (Figure 1A). The mutation, c.3G > A (p.Met1Ile) was previously shown to be associated with variants contributing to HCM (rs397516045) 10. However, the *OBSCN* gene mutation at c.15652_15652del (p. Leu5218fs) was a novel finding. The results of homology comparison showed that the location of *obscn* gene mutation sites was highly conserved in the whole species (Figure S2).

### Patient-derived induced pluripotent stem cell (iPSC) and iPSC based cardiomyocytes (iPSC-CMs)

We successfully generated 3 iPSC lines from PBMCs obtained from patients with ARVC. We selected and used iPSC colonies with human embryonic stem cell (hESC) morphology (Figure 1D). Stable iPSC lines were subcultured and cryopreserved for nearly over 35 generations. Subsequent experiments utilized iPSC lines past the 20th generation. All tested iPSC lines exhibited similar expression levels of the endogenous *POU5F1* and *NODAL* pluripotency-related genes observed in hESCs based on the results of quantitative real-time polymerase chain reaction (qRT-PCR) analysis (Figure 1E). Concomitantly, iPSCs were stained for pluripotency markers (SSEA-4 and TRA-1-60) detected by flow cytometry (Figure 1F) that retained long telomeres and normal karyotypes (Figure 1G). The pluripotency of iPSCs was further confirmed following subcutaneous injection in SCID mice. All iPSC lines tested produced teratomas, whose structures and tissues, including glandular structures, cartilage, and neuroepithelium were derived from 3 embryonic germ layers (Figure 1H-I).

**Figure 2:**
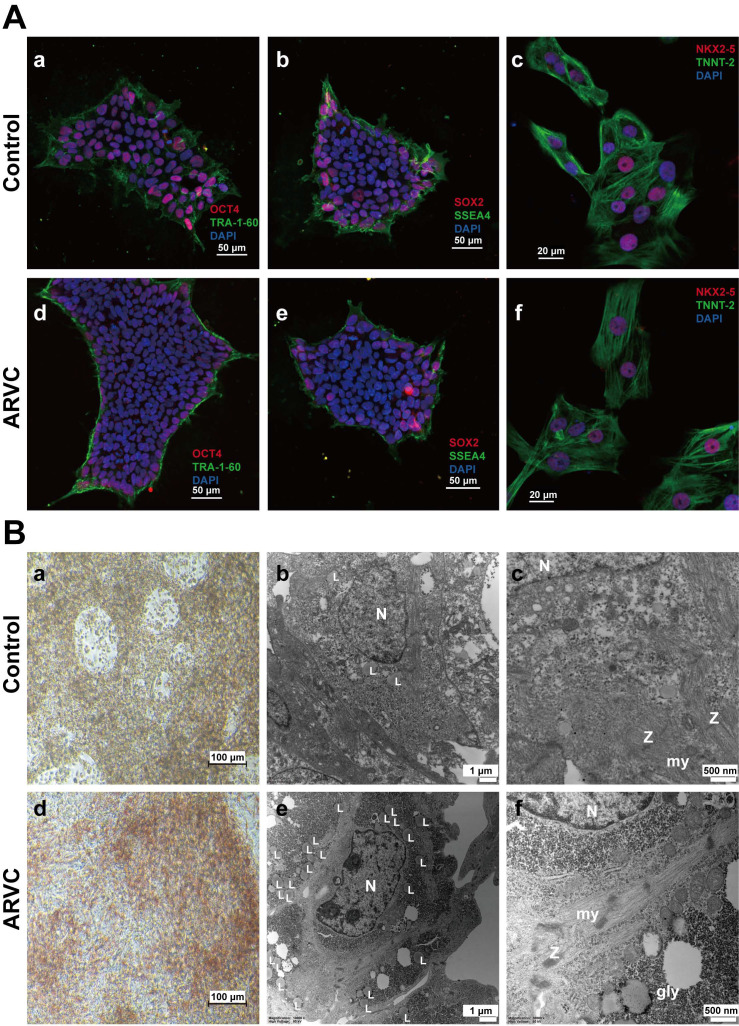
** Identification of induced pluripotent stem cell based cardiomyocytes** (A) Immunofluorescence staining of Oct4 (a)(d), TRA-1-60 (a)(d), SOX2 (b)(e) and SSEA4 (b)(e) in iPSC of ARVC or control groups. Positive staining of Oct4 (red) and SOX2 (red) within the cell nuclei, and positive staining of TRA-1-60 (green) and SSEA4 (green) on the cell membrane. Immunofluorescence staining of TNNT-2 (c)(f) and NKX2.5 (c)(f) in iPSC-CMs of ARVC or control group. Positive staining of NKX2.5 (red) within cell nuclei, and positive staining of TNNT-2 (green) on the cell membrane. Cell nuclei were counterstained with 4',6-diamidino-2-phenylindole (DAPI, blue). Scale bars of 50μm (images on the upper left); and 20μm (images on the upper right). (B) Images of Oil Red O staining images of CMs from ARVC and the control groups (a)(d). Most ARVC-iPSC-CMs were positivity stained for lipid droplets (red) within cells, whereas only a small number of control cells were stained positive for lipids. Scale bar: 100 μm. TEM images of iPSC-CMs containing lipid droplets in cell cytoplasm (b and e, 10000×) and the ultrastructure of CMs (c and f, 30000×). Small-scale lipid droplets (L) observed close to the nucleus (N) in iPSC-CMs from a control subject (b) and the ARVC patient (e). iPSC-CMs from the ARVC patient appear to possess a larger number of lipid droplets in comparison to control cells. Myofibrils (my) are organized in different sarcomeric structures according to the description of Z-bands (Z). Glycogen masses (gly) can be seen in the cytoplasm. N, nucleus.

All iPSCs uniformly expressed the OCT4, stage-specific embryonic antigen-4 (SSEA-4), SOX2, and TRA-1-60 pluripotency markers, as determined by immunofluorescence staining (Figure 2A). Small-scale clusters of iPSC-CMs obtained from both groups exhibited positive staining for NK2 homeobox 5 (NKX2.5) and troponin T2 (TNNT-2), as well as displayed CM recognition (Figure 2A).

### Oil Red O staining and ultrastructural analysis using Transmission Electron Microscopy (TEM)

Compared with control iPSC-CMs, positive staining for Oil Red O in ARVC-iPSC-CMs was significantly higher (red) (38.44 ± 5.4% *vs.* 8.58 ± 0.59%; n = 6, *P* = 3.42e-05). Lipid droplets were graded in cells in a high magnification range (20×) (Figure 2B). Clustered positive staining of lipid droplets (red) was observed in ARVC-iPSC-CMs by Oil Red O staining, while only sparse positive staining was observed in control-iPSC-CMs (Figure 2B). No significant differences were noted in the calculated intensity of Oil Red O staining between ARVC-iPSC-CMs and the control (143.18 ± 11.08 *vs.* 143.85 ± 4.59 a.u.; n = 6, *P* = 0.90).

TEM analysis of iPSC-CMs of the 2 groups revealed ultrastructural binding of myofibrillar-based cells along with clearly visible Z-bands (Figure 2B). By examining 6 images per group, a significant increase in the absolute numbers of lipid droplets was demonstrated in each of the images of the ARVC group compared to the control iPSC-CMs (40 ± 4.9 compared with 11 ± 3.2; *P* = 1.17e-06). Moreover, the Z-bands organization in ARVC-iPSC-CMs was more irregular, and compared with the Z-bands in the control group; their thickness and pleomorphism were also increased.

### Electrophysiological description of single ARVC-iPSC-CMs

**Figure 3:**
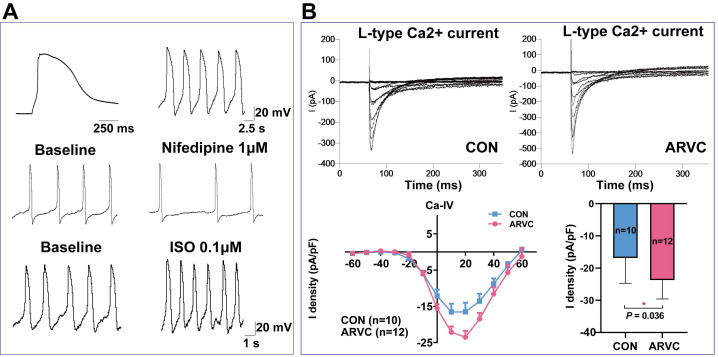
** Electrophysiological characterization of ARVC-iPSC-CMs** (A) Action potential profile of a typical ventricular-like cell and its corresponding reaction to 1μΜ nifedipine and 0.1μM of isoproterenol (ISO). (B) Representative L-type calcium current trace and I-V curve in voltage-clamp pattern resulting from a holding potential of -60 mV and test pulses ranging from -60 to +60 mV.

Following exposure to the isoproterenol β-agonist, and the nifedipine calcium channel antagonist, most ARVC-iPSC-CMs (70%) exhibited a ventricular-like AP distribution, as well as predicted responses (Figure 3A). Both groups of cells showed similar proportions of spontaneous cell contraction. In the presence of nifedipine (1 μM), the rate and magnitude of contraction were reduced in two groups, but their rates were multiplied in the coculture of isoproterenol (0.1 μM). According to voltage-clamp studies, significant differences were observed between the two groups (*P* = 0.036). The L-type calcium current (ICaL) peak density of CMs in the control group was -16.8 ± 6.8 pA/pF (mean ± SEM, n = 10), whereas the ICaL peak density in the ARVC group was -23.7 ± 5.9 pA/pF (mean ± SEM, n = 12) (Figure 3B).

### Identification of differentially expressed genes (DEGs) in ARVC-iPSC-CMs

**Figure 4:**
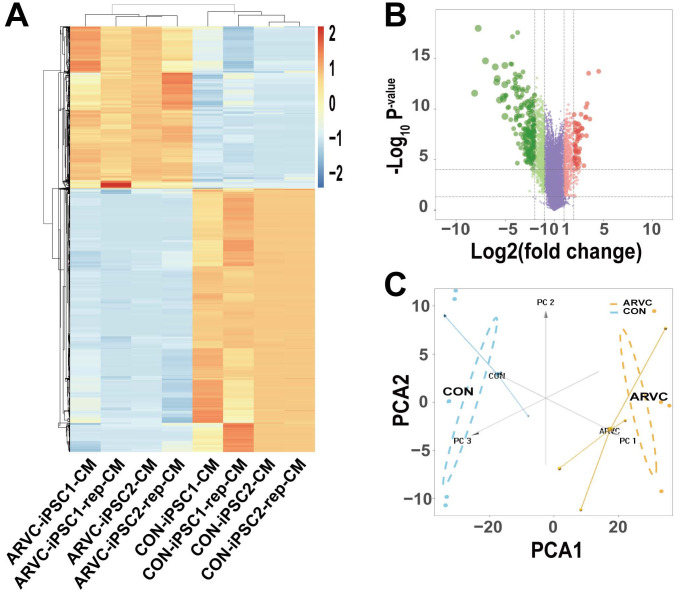
** Identification of differentially expressed genes (DEGs)** (A) Heatmap showing DEGs of ARVC and control groups. Samples are classified in columns, and genes are classified in rows. (B) Volcano plot of all DEGs. Purple dots stand for genes with no significant differences in their expression between the 2 groups. Green dots represent downregulated genes, and red dots represent upregulated genes. (C) Trajectory plots of PCA scores displaying differences arising in the ARVC and control groups. Blue point, control group; yellow point, ARVC group.

We obtained the gene expression profiles of four samples each of ARVC-iPSC-CMs and control iPSC-CMs. As illustrated in the volcano plot (Figure 4B) 270 DEGs (64 upregulated and 206 downregulated) were selected under the specified conditions (adjustment threshold *P* < 0.05 and |log2FC|>2) from 30 931 genes within the dataset (Table S1). Their heatmap revealed that DEGs could be used to distinguish the groups (Figure 4A). Further, the score trajectory plots from the principal component analysis (PCA) of ARVC failed to show a substantial overlap with the control group profiles, indicating obvious differences in the parallel PCA plots (Figure 4C), warranting further analysis.

### Identification of DEG pathways in ARVC-iPSC-CMs by functional enrichment analysis

**Figure 5:**
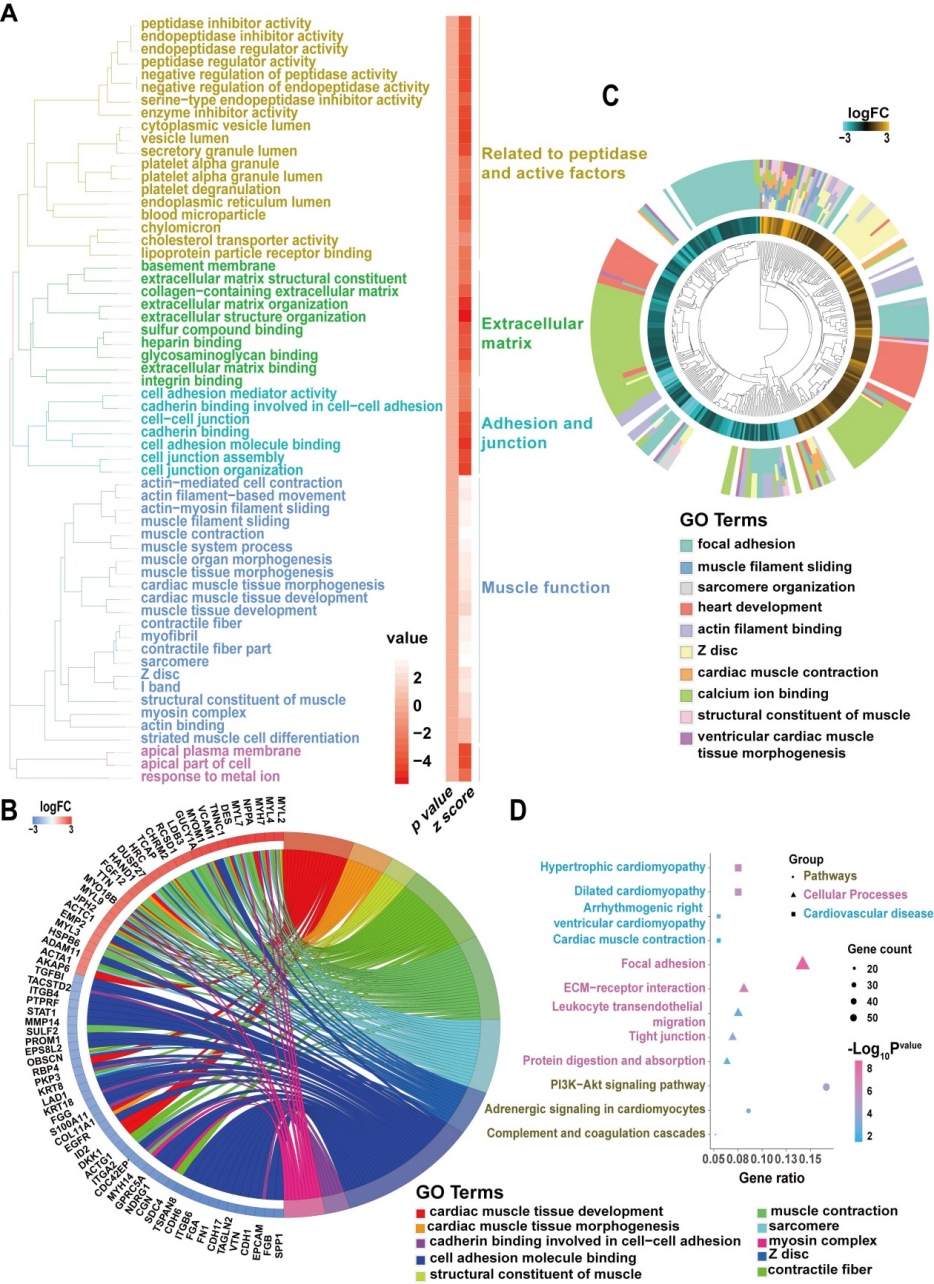
** Candidate driver genes and pathways in ARVC-iPSC-CMs** (A) GO and pathway summary of DEGs in the ARVC group. GO and pathway categories divided according to the functional theme. (B) GOChord plot shows the genes related to over 3 pathways and in association with the ARVC state, contributing to the enrichment, organized according to the order of their expression level. *OBSCN* is at the forefront. (C) GOCluster plot explaining the relevance between DEGs of high association with ARVC and their related GO terms. For all genes, their high/low logFC values are interpreted by brown (turquoise) rectangles. These genes are mainly focused on calcium ion binding (green), heart development (red) and focal adhesion (cyan). (D) Chart revealing KEGG enrichment of DEGs in signaling pathways. The Y-axis label stands for the pathway, and the X-axis label represents the gene ratio (gene ratio = number of DEGs improved in the pathway/number of all genes within the background gene set). Squares, triangles, and circles represent pathways, cellular processes, and cardiovascular diseases, respectively. Size and color stands for the number of enriched DEGs of bad prognosis sets in the pathway and the significance of enrichment.

Our analysis showed that the GO and pathway category analyses grouped the DEGs in ARVC samples into 6 categories according to functional theme (Related to peptidase and active factors, extracellular matrix, adhesion and junction, muscle function) (Figure 5A). The GOChord plot (Figure 5B) shows 70 DEGs related to the 3 pathways associated with the ARVC state, exhibiting enrichment in the presented order level. The GOCluster plot (Figure 5C) demonstrates the interaction between GO term genes and clusters. Furthermore, the biological stages of DEGs revealed that they were mainly focused on calcium ion binding (green), heart development (red), and focal adhesion (cyan). As per the KEGG pathway analysis, the ARVC group's DEGs were greatly enriched in 3 groups, including pathways, cellular processes, and cardiovascular diseases (Figure 5D), which included ARVC, cardiac muscle contraction, and hypertrophic cardiomyopathy, as well as dilated cardiomyopathy. The cellular processes comprised of five categories of focal adhesion, ECM-receptor interaction, leukocyte trans-endothelial migration, tight junction, protein digestion, and absorption. Besides, potential pathways included the PI3K-Akt signaling pathway, adrenergic signaling in CMs, and complement and coagulation cascades. Thus our results of the functional enrichment analysis identified processes involved in focal adhesion and structure.

### Structural disorders and decreased expression of obscurin and its anchoring protein in ARVC-iPSC-CMs

**Figure 6:**
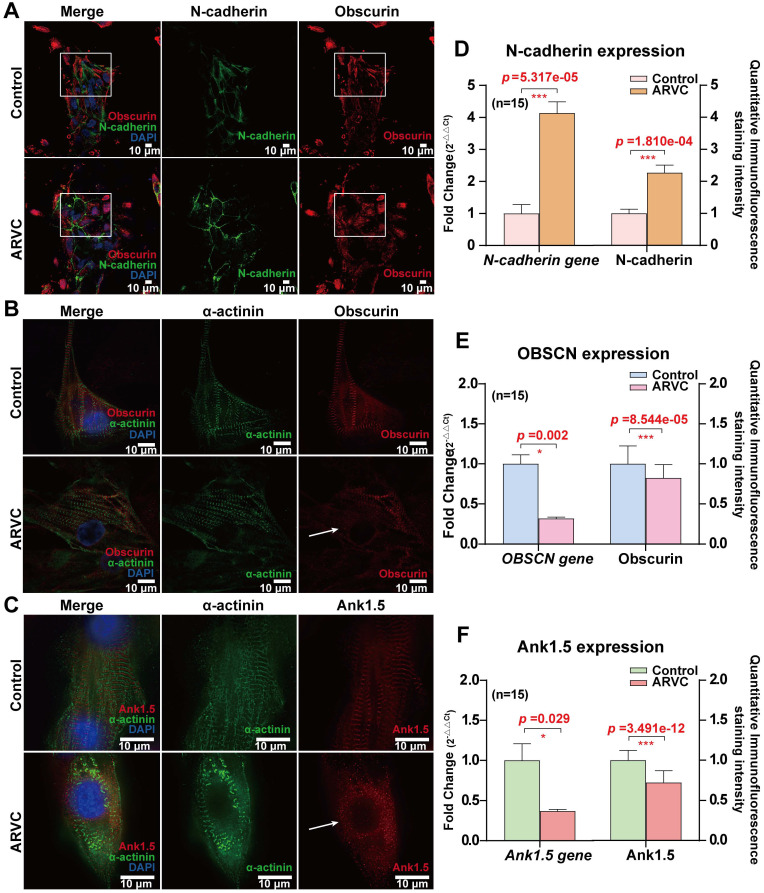
** Mapping of junctional protein N-cadherin and obscurin-binding ankyrin 1 (Ank1.5) for exploring the influence of *OBSCN* mutations on subcellular structures** (A) There is no significant difference in the localization of N-cadherin (green) between the two groups, but the expression level of N-cadherin is increased in the disease group: obscurin (red) is located at the M-line level, cross distributed at approximately equal intervals (60×; oil mirror). The distribution of obscurin markers in the ARVC group is disordered (white box). (B) Observation under an ultra-high-resolution microscope reveals that in the control group actin (green) is located at the Z-disk level, whereas obscurin (red) is located at the M-line level, cross distributed at approximately equal intervals, with a spacing of about 200 nm (60×; oil mirror). The distribution of obscurin markers in the ARVC group is disordered (white arrow). (C) Ank1.5 binds to the ankyrin binding domain of obscurin in the sarcoplasmic reticulum. In fully developed striated muscle cells, ank1.5 is mainly located at the M-line level (red). In the control group, actinin (green) and ank1.5 (red) show regular interval distribution. In contrast, the ank1.5 (red) distribution is disordered and scattered in the ARVC group (60×; oil mirror). (D, E, F) Quantitative calculation of the immunofluorescence staining intensity and expression levels of junctional protein, mutant protein, and its anchor protein. In the ARVC group, the level of expression of *OBSCN* and *ANK1.5* genes, as well as the immunofluorescence signal intensity are significantly decreased (all *P<* 0.05), but significantly increased of the junctional protein N-cadherin (*P<*0.05).

To examine the distribution of obscurin, we co-stained it with the junctional protein N-cadherin. There was no significant difference in N-cadherin's localization between the two groups (Figure 6A), but its expression level was increased in ARVC-iPSC-CMs (Figure 6D). Both actinin and obscurin are known to be sarcomere-associated structural proteins. The former was reported to be located in the Z disc of the striated sarcomeric structure of the muscle fiber 11, whereas the latter was shown to be mainly located at the M-line level of mature striated muscle cells 12, 13. Accordingly, their spaced cross-distribution has been regarded as a hallmark of mature striated muscle fibers. Ankyrin1.5 (Ank1.5) is a muscle-specific isoform of ankyrin1, an anchor protein that binds to obscurin's ankyrin binding domain in the sarcoplasmic reticulum.

To quantitatively compare the expression of the mutant obscurin protein and its anchor protein, a monolayer of cardiomyocytes was prepared from concentrated cell clusters and quantitative analysis was performed to determine the concentrations of both proteins. Immunofluorescence staining of the monolayer cultures of ARVC and control iPSC-CMs exhibited similar staining levels for the cardiac sarcomeric marker (Figure 2A) and α-actinin (Figure 6B-C). Under an ultra-high resolution microscope, we could observe that α-actinin (green, Figure 6B-C) was located at the Z-disk level, whereas obscurin (red, Figure 6B) was located at the M-line level in the control group, cross-distributed at approximately equal intervals, with a spacing of about 200 nm (Figure 6B). However, the distribution of obscurin (red, Figure 6B) and its anchoring protein Ank1.5 (red, Figure 6C) markers were disordered in the ARVC group. The mRNA expression levels of the *OBSCN* and *ANK1.5* genes, as well as immunofluorescence staining of their products were remarkably reduced in the ARVC group (all *P <* 0.05, Figure 6E-F).

### Quantification of the expression of the mutant protein and related proteins in CMs

**Figure 7:**
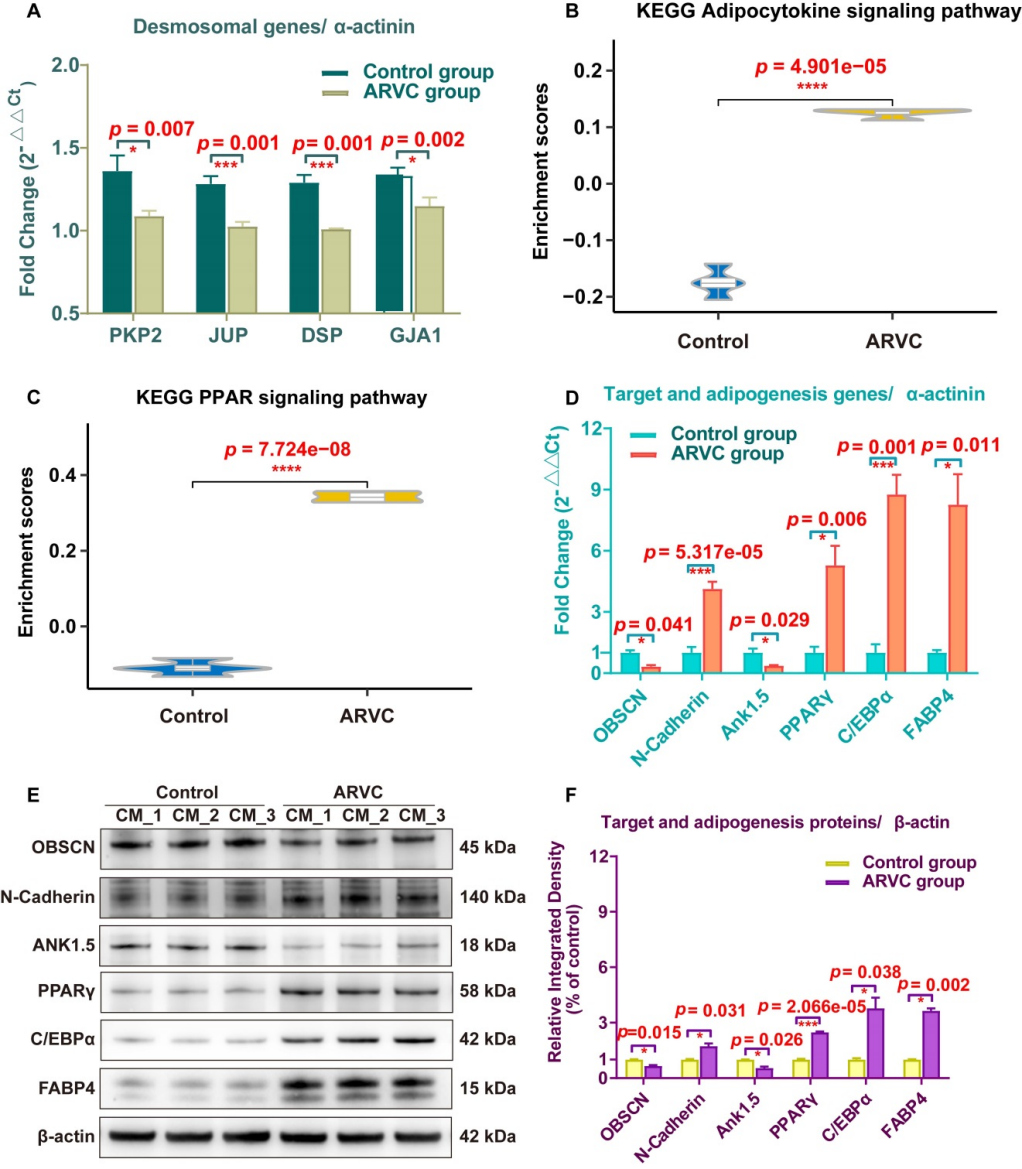
** Expression analysis of specific genes and proteins in iPSC-CMs** (A) qRT-PCR was performed to survey the expression of plakoglobin (*JUP*), desmoplakin (*DSP*), plakophilin2 (*PKP2*), connexin43 (*GJA1*), and α-actinin (*ACTN1*) genes in iPSC-CMs of both groups. (B, C) The enriched scores of adipocytokine and PPAR signaling pathways were higher in the ARVC group by the ssGSEA method. (D) Comparison of mRNA results of target and adipogenesis pathway-related genes (*OBSCN, N-Cadherin*, *Ank1.5*, *PPARγ*, *C/EBPα* and *FABP4*) in two groups. (E, F) Detection of target and adipogenesis pathway-related proteins in iPSC-CMs by Western blots. β-actin was used as a loading control. Bands were quantified with Image J software. One asterisk indicates *P* <0.05, and three asterisks are shown as *P* <0.001 compared with the control group.

To validate the expression of the target mutant protein, desmosomal proteins, anchoring protein, junctional proteins, and adipogenesis- and adipocytokine pathway-related proteins, we used iPSC-CMs samples of the two groups. The mRNA expression of the total 10 genes (*PKP2*, *JUP*, *DSP*, *GJA1*, *OBSCN*, *N-Cadherin*, *Ank1.5*, *PPARγ*, *C/EBPα,* and *FABP4*) was examined using qRT-PCR in 6 samples from each group. The transcription of *PKP2*, *JUP*, *DSP*, and *GJA1* in ARVC-iPSC-CMs were significantly reduced compared with those of control-iPSC-CMs (all *p* < 0.05, Figure 7A). Gene expression was standardized to α-actinin (Figure 7A-D). The adipocytokine and PPAR signaling pathways had higher scores in the ARVC group (all *p* < 0.05, Figure 7B-C); qRT-PCR and Western blotting were used to validate some of these results (Figure 7E-F). The qRT-PCR analysis showed that the mRNA levels of mutant gene *OBSCN* and its anchoring protein gene *Ank1.5* in ARVC-iPSC-CMs were about one-third of the control-iPSC-CMs (Figure 7D). However, other target- and adipogenesis pathway-related genes, *N-Cadherin*, *PPARγ*, *C/EBPα,* and *FABP4* increased by 4.13-, 5.28-, 8.77-, and 8.26-fold, respectively, in the ARVC group compared with the control group, (Figure 7D). The proteins of above genes were also changed significantly in the ARVC group, with the levels of OBSCN and Ank1.5 decreased by 0.65- and 0.55- fold, and levels of N-Cadherin, PPARγ, C/EBPα, and FABP4 increased by 1.73-, 2.48, 3.78, and 3.64- fold, respectively, compared with the controls (Figure 7E-F).

## Discussion

To the best of our knowledge, this is the first study describing the generation of iPSC-CMs isolated from an ARVC patient carrying a novel mutation in the *OBSCN* gene. Our data provided a detailed demonstration of the significant phenotypic differences between ARVC- and control-iPSC-CMs. Ultrastructural analyses of CMs by TEM confirmed the substantial baseline differences between ARVC- and control-iPSC-CMs, as indicated by lipid droplets, increased pleomorphism, and irregular Z-bands. Compared with the control group, ARVC-iPSC-CMs exhibited an increase in the intracellular calcium current in our voltage-clamp studies. Subsequently, functional enrichment analysis of pathways showed that the differentially expressed genes in the ARVC group were mainly associated with focal adhesion and structure, and adipocytokines and PPAR signaling pathways. Moreover, our results from ultra-high resolution microscopy, qRT-PCR analysis and Western blotting confirmed that the mutant OBSCN protein and its anchor Ank1.5 protein showed structural disorder and decreased expression, but increased expression of N-Cadherin was observed. Meanwhile, the gene expression of other desmosomal proteins in ARVC-iPSC-CMs was also decreased whereas some of the adipogenesis pathway-related proteins (PPARγ, C/EBPα, and FABP4) were increased.

### Relevance to clinical disease

Analysis of the pathways affected in human ARVC-iPSC-CMs carrying the *OBSCN* mutation mainly identified components involved in adhesion molecule binding and the PI3K-Akt signaling pathways. These observations were consistent with results from study in mouse models and rat heart myocytes as models of myocardial infarction 14, suggesting the possibility of employing iPSC-CMs as a cell model for the study of novel gene mutations in patients with ARVC.

Our data furnished information on the disease in 2 ways: (i) Microscopic analysis provided evidence of increased lipid droplets content in the ARVC-iPSC-CMs compared with the control group. We observed the activation of adipocytokines and PPAR signaling pathways that might explain the production of adipose fibrosis. Moreover, the Z-bands organization in ARVC-iPSC-CMs was more irregular with increased thickness, and more pleomorphic than the Z-bands observed in the control group. Electrophysiological characterization showed that the ICaL density of ARVC-iPSC-CMs was increased, and this imbalanced ICaL status might be one of the causes of arrhythmia. (ii) Functional enrichment analysis indicated the involvement of genes associated with focal adhesion and structure, providing a possible explanation for the mutant protein's structural abnormalities and its anchor counterpart observed in ARVC-iPSC-CMs under ultra-high-resolution microscopy. The gene expression of desmosomal proteins in ARVC-iPSC-CMs was also reduced, possibly partially contributing to the disease progression.

Based on these clinical studies of patients, we plan to investigate the correlation between novel gene mutations and ARVC phenotypes. It would be worth exploring other ARVC patients carrying various mutations and different clinical characteristics.

### iPSC-CM model of ARVC

ARVC refers to rare cardiomyopathy with both electrophysiological and histopathological abnormalities. Human studies and animal models have shown that arrhythmias of ARVC occur earlier than histopathological abnormalities 15, 16. Data from human terminal myocardial samples obtained from patients with ARVC have provided crucial insights into structural mutations and their correlation with the prevalence of the disease, as well as the complex binding interactions of proteins in the interconnected discs 17. Nevertheless, unraveling the mechanisms involved in cellular electrophysiology and cardiac ion channels functions require the study of living cells. Based on the analysis of exome sequencing, the National Human Genome Research Institute 18 reported that most of the mutations in iPSCs come from rare genetic events in the terminally differentiated parental cells and that the reprogramming process would not increase the probability of genetic mutations. Therefore, patient-derived iPSCs, carrying specific mutations, could be frozen, stored, and employed as an *in vitro* model of the disease to provide structure-based and functional data for developing novel therapeutic applications.

Previously, the iPSC-CM model was used to perform patch-clamp studies in a patient with ARVC carrying a mutation in the *DSG2* gene, where an abnormal action potential and multiple ion channel currents were detected 19. Animal models studies demonstrated that an increase in the calcium storage in the myocardial sarcoplasmic reticulum of mice carrying the r4344q mutation of the *OBSCN* gene resulted in the instability of the myocardial calcium current. Long-term stimulation under an unstable calcium current might cause ventricular remodeling, secondary cardiac hypertrophy, or ventricular enlargement. In this study, a deletion mutation in the *OBSCN* gene in patients with ARVC resulted in a frameshift mutation at position 5218 and its corresponding protein domain. In this study, quantitative detection in ARVC-iPSC-CMs performed by patch-clamp showed a significant increase in ICaL, suggesting abnormal calcium circulation, partially explaining the correlation between the instability of the myocardial calcium current and ventricular enlargement; however, the specific mechanism was unknown.

### Mutations in *OBSCN* and ARVC

The patient carried a novel heterozygous mutation in *OBSCN* (c.15652_15652del) in this study. In the “1000 genomes” database, ClinVar and the Exome Variant Server databases, including both normal and pathological genomes, the frameshift variant of *OBSCN* in our study was absent. The deletion mutations of *OBSCN* in the conservation analysis of species comparison showed that they would affect fully conserved residues (Figure S2). We, therefore, hypothesized *OBSCN* (c.15652_15652del (p. L5218fs)) to be a potential disease-causing mutation. Several *OBSCN* mutations have been described to be associated with hypertrophic cardiomyopathy (HCM), dilated cardiomyopathy and left ventricular non-compaction, indicating variations in the penetrance and phenotypic representation of the disease 20-22. In a study of 30 index patients with end-stage heart failure, mutations in *OBSCN* were identified in 5 subjects (17%) 21. similarly, heterozygous mutations in *OBSCN* were identified in 6 of 74 unrelated individuals with hypertrophic cardiomyopathy, and in 3 of 10 patients with left ventricular densification 23.

The patient in our study, a Chinese national clinically diagnosed with ARVC according to the criteria set by the Task Force 24, developed this novel heterozygous mutation in *OBSCN* that might be of relevance to the pathogenesis of ARVC (whole genome sequencing did not detect other genes known to be associated with ARVC). The T-wave inversion and epsilon waves clinically manifested in the electrocardiogram and the right ventricular abnormality on the echocardiography observed in our patient were similar to the Italian-based clinical characteristics portrayed in patients with ARVC also carrying mutations in *OBSCN* 21, 23. We also determined that the *OBSCN* mutation was related to the decrease of protein expression level of obscurin, pointing to haploinsufficiency as a possible disease-causing mechanism. Thus, *OBSCN* mutations may be significant contributors to the ARVC burden. So far, *OBSCN* mutations have been rarely detected probably because of the large size of the gene.

Although the detection of protein level in this study was aimed at a fragment of the target protein, the antibody specificity of the protein included the position before and after the mutation, and its result of western blot could indicate the change of protein level. In this study, the patient's family also carried the *MYBPC3* gene mutation c.3G > A(rs397516045), which might be associated with HCM, but heterozygote of this mutation was clinically unaffected with a normal echocardiography in the previous study 10, which was consistent with our study. Sanger sequencing showed that the proband's father and son carried a single *MYBPC3* c.3G > A (p. Met1Ile) mutation and exhibited normal heart function. Other family members without *OBSCN* mutations of the proband were clinically unaffected with normal ECG and echocardiography. Therefore, *OBSCN* mutation was categorized as a possibly pathogenic unclassified type 3 variant.

Our functional enrichment analysis pointed to pathways involved in focal adhesion and structure. Therefore, we focused on the distribution and mutational status of structure- and adhesion-related genes. Interestingly, although our patient did not have a known mutation in the anchor protein and junctional protein, the gene and protein expression levels of the ANK1 and N-cadherin as well as the intensity of immunofluorescence staining for ank1.5 were decreased and for N-cadherin were increased. It was consistent with the decreased mRNA and protein content of the mutant protein OBSCN (Figure 7E-F). Herein, we demonstrate that reduced expression and disrupted localization of the mutant protein OBSCN was accompanied by increased expression of junctional proteins like N-cadherin and reduced expression and perturbed localization of its anchoring protein ank1.5. Also, altered expression levels of the junctional protein N-cadherin were accompanied with similar changes in the transcript levels (Figure 6A-D), consistent with a previous report 25, suggesting that obscurin may play a key role in maintaining and stabilizing proteins in the cell-cell junctions, such as the scaffolding role of obscurin in striated muscle cells 8, 26. Obscurin and ankyrin1 are known to be structural proteins connecting the M-line of the sarcomere with the sarcoplasmic reticulum, which binds to the obscurin protein and promotes the longitudinal extension of the sarcoplasmic reticulum along with the muscle fiber. Also, obscurin and ankyrin1 are known to share mutual molecular interactions 7, 8. It is possible that the reduced levels of OBSCN led to the reduced colocalization of ankyrin1, although the patient did not carry a mutation in the ANK1 gene. Additionally, the distribution of ank1.5 in control-iPSC-CMs showed regular interval distribution with α-actinin, suggesting that the sarcoplasmic reticulum extended to the M-line level. However, in ARVC-iPSC-CM, the distribution of ank1.5 was scattered in dots and not correlated with the distribution of α-actinin, suggesting that the development sarcoplasmic reticulum was impaired in patients with ARVC.

Sarcoplasmic reticulum is considered an important calcium ion storage in CMs. Accordingly, the sarcoplasmic reticulum extending into the sarcomere makes the excitation-contraction coupling of CMs fast and efficient. Therefore, impairment in the distribution of the sarcoplasmic reticulum in patients with ARVC might indirectly affect excitation-contraction coupling. Since the pathological hallmark of ARVC is replaced by fibrous and adipose tissue 1, we compared the enrichment scores for adipogenesis and adipocytokine-related pathway from the KEGG pathway database. The adipocytokine signaling pathway and PPAR signaling pathway scores were higher in ARVC-iPSC-CMs than in Control-iPSC-CMs (Figure 7B-C), indicating that the pathways were activated. The pathways related to adipogenesis and adipocytokines are not shown in the GO results (Figure 6B-C) because many differentially expressed genes were enriched in pathways related to structure and adhesion. Our follow-up experiments have also verified that it is consistent with the phenotypic changes of increased fibrous and adipose cells in the ARVC group.

## Study limitations

Our study demonstrated that iPSC-CMs originating from an ARVC patient exhibited specific disease phenotypes. We have showed that a mutation in the *OBSCN* gene was linked to the pathogenicity of ARVC. However, this observation needs to be confirmed by gene editing, which would repair the gene mutation, reverting the iPSC-CM phenotype to normal. Also, the iPSC-CM research model has its own limitations. Although iPSC-CMs retain the genetic information of the patient and exclude the influence of environmental factors on the disease, possible differentiation of cardiac myocytes in the human body should also be considered. Cardiac sinoatrial nodes, conduction beams, and ventricular myocytes exhibit different electrophysiological characteristics and specific distributions, and the generated iPSC-CMs are a mixture of these heterogeneous cardiac myocytes.

## Conclusion

Herein, we have identified that the OBSCN gene causes ARVC. A frameshift mutation in OBSCN caused its disrupted localization and decreased expression of the anchoring protein ankyrin1.5 in ARVC-iPSC-CMs. Although little is known about the function of *OBSCN*, it could increase L type calcium currents and also accumulate lipids that might be associated with the PPARγ pathway. This may explain the fibrofatty replacement of the myocardium in ARVC patients and lead to dysrhythmia in calcium channel-related myocardial contraction. Future directions include functional studies of the OBSCN protein and suggest that ARVC progression may be attenuated by therapeutic modulation of OBSCN expression.

## STAR Methods

### Clinical details and genetic profile of patients

A 45-y-old Chinese woman who had 3 syncopal episodes on the same day, survived after successful defibrillation for a cardiac arrest. When symptomatic, the cardiac monitor recorded wide-QRS complex tachycardia. Her electrocardiograph showed sinus rhythm, first degree atrioventricular block, right bundle and left-posterior branch block, T-wave inversions in leads V1 to V3, as well as epsilon waves (Figure 1B). Echocardiography showed moderate dilatation of the right ventricle with a reduced motion of the right ventricular free wall, whereas the left ventricular size and function were normal (left ventricular ejection fraction was 62 %). She had no previous history or symptoms of heart disease. However, she came from a family with a history of sudden cardiac death (2 sisters of her grandfather died suddenly from cardiac arrest at the age of 40 and 43). Accordingly, she was diagnosed with ARVC based on the International Task Force criteria (2 major; 1 minor, Table S2.) 24. One week after the onset, the patient received a cardioverter defibrillator (ICD) implant. During the next 9 mo, the ICD system recorded 3 episodes of ventricular tachycardia, which were converted by electric shock (Figure S1). Whole exome sequencing revealed a heterozygous mutation in the *OBSCN* gene (c.15652_15652del (p. L5218fs); Figure 1C), a frameshift mutation located at a splice site that might cause exon skipping. No other pathogenic mutations were detected in sarcomere, ion channel, or other ARVC-related genes.

### Whole exomes and Sanger sequencing analyses

Whole exomes and sanger sequencing were performed on DNA samples from familial proband and family members for genotype confirmation.

### Generation of induced pluripotent stem cells

All studies involving humans were approved by the Peking Union Medical College Hospital and informed consent was obtained from patients. Peripheral blood was obtained from patients with ARVC and age- and gender- matched control volunteers with no clinical history or indication of ARVC. PBMCs were isolated by density gradient centrifugation 27. To facilitate reprogramming by increasing the infection efficiency of PBMCs, cells were infected with lentiviruses containing octamer-binding transcription factor 4 (OCT4), Kruppel-like factor 4 (KLF4), SRY-box transcription factor 2 (SOX2), and MYC proto-oncogene (c-MYC) using a reprogramming Kit (CytoTune™-iPS 2.0 Sendai Kit, A16517, Thermo Fisher Scientific, Waltham, MA, USA). To stimulate spontaneous differentiation into 3 germ layers, iPSCs were inoculated in differentiation medium (Dulbecco's modified Eagle's medium supplemented with 20% knockout serum, 0.1 mM nonessential amino acids, 2 mM L-glutamine, 50 μM 2-mercaptoethanol; Thermo Fisher Scientific) on ultra-low attachment surface 6-well plates (Corning Incorporated, Corning, State of New York, USA). Half of the medium was replenished daily. On day 4, embryoid bodies (EBs) were harvested and seeded on Matrigel-coated slides and differentiated for another 7 d.

### Induced pluripotent stem cells and cardiac differentiation

The pluripotency of iPSCs could be visualized by employing immunofluorescence staining, providing a measure of gene expression in human pluripotent cells. Briefly, iPSCs (well plate coated with Matrigel; Corning 354277, Corning Incorporated, Corning, State of New York, USA) were seeded as small clumps of cells in TeSR-E8 (Stemcell 05990, STEMCELL Technologies Inc, British Columbia, Canada) containing 10 μM ROCKi (Y27632, Selleck Chemicals, Houston, USA) 4 d before differentiation. The medium was changed to TeSR-E8 without ROCKi 1 day later and cells were further cultured for another 3 d. For cardiac differentiation, the STEMdiff Cardiomyocyte Differentiation Kit (05010, STEMCELL Technologies Inc, British Columbia, Canada) was applied to iPSC colonies according to the manufacturer's instructions. The principle of producing cardiomyocytes is consistent with the previous literature; that is, regulating wingless signal transduction 28. All CMs used in subsequent experiments were allowed to differentiate for 4 to 5 weeks to ensure similar maturation levels.

### Teratoma assay

Using classic teratoma generation assays 29, cells (2-4 × 10^6^) were subcutaneously injected in the flank area of 4-wk-old immunocompromised SCID mice (Beijing Vital River Laboratory Animal Technology Co., Ltd., Beijing, China). Tumor growth was monitored weekly by palpation. Mice were euthanized when tumors reached a size ≥ 2 cm^3^ after 8 wk. All animal experiments were approved by the Animal Ethics Committee of the Peking Union Medical College Hospital.

## Description of iPSC-CMs

### Recordings of single-cell patch-clamp

To record action potentials, iPSC-CMs were enzymatically digested into single cells 2 d in advance, and adhered to 0.1% glass covered with coverslips. Micropipettes were prepared from 1.5 mm thin-walled glass (WPI Instruments, Sarasota, USA), using a filament micropipette puller (P-97, Sutter Instruments, Novato, USA). Single beating CMs underwent whole-cell patch-clamp at 32-35 °C by employing an EPC-10 patch-clamp amplifier (HEKA Electronics, Lambrecht, Germany) belonging to a RC-26C recording chamber (Warner Instruments, Hamden, USA). Whole-cell L-type calcium current recordings were performed with the help of an EPC-10 patch clamp amplifier controlled by the PULSE software (HEKA Electronics) and fixed upon the position of an inverted microscope (Nikon, Tokyo, Japan). Next, data were captured using the PatchMaster software (HEKA Electronics), and digitalization was done at 1.0 kHz.

### Immunofluorescence staining and confocal microscopy guided by iPSCs, junctional protein, cardiac contractile and anchoring proteins

Pluripotency markers of iPSCs were determined by using Human Embryonic Stem Cell Marker Panel (1:1000; ab109884, Abcam plc., Cambridge, UK). The structural composition of CMs could be identified by the presence of α-actinin cardiac contractile proteins as determined by immunofluorescence staining. To this end, we used mouse monoclonal anti-α-actinin (1:500; A7811, Sigma Chemical Co., St. Louis, USA), anti-obscurin (1:500; hpa021186, Sigma), anti-N-Cadherin (1:1000; ab19348, Abcam) and anti-ank1.5 (1:500; ARP42566_T100, Aviva Sysbio, San Diego, USA) antibodies. All generated iPSC-CMs were counterstained in the context that antibodies against α-actinin were used to validate their identity and structural composition. The steps of antibody staining in the experiment were consistent with the previous research steps 30. Stained beating EBs and monolayer cells were observed using laser-scanning confocal microscopy (LSM 710, Carl Zeiss, Jena, Germany) and images were captured with the Delta Vision OMX SR imaging and analysis system of the ultra-high resolution microscope.

### Transmission Electron Microscopy (TEM)

The 4 to 5 wk-old iPSC-CMs differentiated from PBMCs, isolated from patients and control subjects, were subjected to TEM (JEM-1400Plus, JEOL, Japan). We chose iPSC-CMs to observe the nucleus/nucleolar, myofilaments, and Z-bands on cell membranes. For the semi-quantitative evaluation of the number of lipid droplets present in the control and ARVC iPSC-CMs, we measured the number of lipid droplets from 6 images and calculated the average number of lipid droplets per image.

### Oil Red O staining

To determine whether cells stemming from patients with ARVC contained more lipid droplets than cells originating from control subjects, we inoculated iPSC-CMs from the ARVC or control groups on gelatin-coated dishes. Subsequently, cell cultures were placed in 4% of paraformaldehyde (158127, Sigma Chemical Co.) at least 10 min. Next, Oil Red O (Sigmao-0625, Sigma Chemical Co.) staining was performed to detect intracellular lipid droplets. Cell cultures were washed with 60 % isopropanol and then stained with freshly prepared Oil Red O working solution at 25 °C for 15 min. Subsequently, cell cultures were rinsed with distilled water, and fixed using an aqueous mountant. Images were captured with a camera attached on a reverse fluorescence microscope (Microscope: Olympus CKX41, Japan).

### Gene Ontology (GO) and Kyoto Encyclopedia of Genes and Genomes (KEGG) analyses

To investigate the potential pathways through which significant differentially expressed genes (DEGs) could impact the relative ARVC state, we performed GO functional enrichment analysis 31 by uploading DEGs onto the Database for Annotation, Visualization and Integrated Discovery (DAVID) 32, as well as visualization of KEGG pathway maps via KOBAS 33. A false discovery rate (FDR) of < 0.05 was used as the cutoff value. We also performed GOChord plotting using the GOplot R package, thus accumulating quantitative molecular data of the GO terms of interest 34, designed to incorporate data obtained through functional annotation enrichment analysis of measured representative information.

### Comparison of enriched adipogenesis pathways

ARVC was characterized by fibrofatty replacement 35; therefore, we extracted a signature of adipogenesis-related pathways containing 67 genes from KEGG (https://www.kegg.jp/). Following a published procedure 36, These gene sets were generated enrichment scores for each pathway in each sample by ssGSEA method, which was calculated as the activated score minus the repressed score. Subsequently, we compared the ssGSEA score of each pathway among the two groups.

### Expression of the target mutant gene and related genes

qRT-PCR was used to determine the mRNA expression levels of the target mutant protein, desmosomal proteins, anchoring protein, junctional proteins and adipogenesis and adipocytokine pathway-related proteins. The following measures were applied to enhance the precision profiling of these genes by excluding potential interference from non-CM contamination: (i) isolation of total RNA from CMs exhibiting a rate of differentiation greater than 85%; and (ii) the expression of desmosomal genes standardized according to the levels of α-actinin. Primers were either obtained from previous studies 25, 37-41 or purchased from the PrimerBank of the Harvard Medical School (https://pga.mgh.harvard.edu/primerbank/index.html) (Table S3).

### Western blotting

iPSC-CMs from the ARVC or control group were lysed in ice-cold RIPA buffer (Beyotime, P0013B, Shanghai, CHN) containing protease inhibitor cocktail (Beyotime, P1048, Shanghai, CHN) and 1 mM PMSF (Beyotime, ST506, Shanghai, CHN) for 30 minutes. Subsequently, the insoluble material of tissue lysates was removed by centrifugation at 12000x g and 4 °C for 15 minutes. According to our previous experimental protocol 42, the protein concentration was measured (Thermo, A53225, Massachusetts, US), normalized to the total protein concentration, cell lysates were resuspended in the SDS sample buffer and then denatured at 98 °C for 5 minutes. Ten micrograms of total protein were fractionated by electrophoresis on preformed 4% to 12% gradient polyacrylamide gels. The proteins were transferred electrophoretically onto a nitrocellulose membrane (Thermo, IB23001, Massachusetts, USA). The membrane was incubated with the appropriate primary antibodies overnight at 4 °C. Proteins were detected by probing Western blots with antibodies specific to β-actin (1:5000, ab8227, Abcam, Cambridge, UK), OBSCN (1:1000, HPA019497, Sigma-Aldrich, Missouri, USA), N-Cadherin (1:1000; 13116, Cell Signaling Technology, Massachusetts, US), ANK1.5 (1:1000, ARP42566_T100, Aviva Sysbio, San Diego, USA), and adipogenesis markers (PPARγ, C/EBP and FABP4, 1:1000, CST, Boston, USA). Following incubation with horseradish peroxidase-conjugated secondary antibodies (Beyotime, A0208 and A0216, Shanghai, CHN), the antigen-antibody complexes were detected with an enhanced chemiluminescence detection reagent kit (Thermo, 34577, Massachusetts, US). Protein bands were visualized with a double-color infrared laser imaging system (LI-COR Biotechnology, Nebraska, US). Densitometry analysis of the gels was carried out using Image J software from the NIH (http://rsbweb.nih.gov/ij/).

### Statistical analysis

Numerical data were shown as mean ± SEM. Comparisons among experimental groups were assessed by the Student's *t*-test (double-tailed) using the Excel software (version 2016 Windows, Microsoft, Redmond, USA). *P<*0.05 was considered significant.

## Supplementary Material

Supplementary data including figures and tables.
