# Supplementary material for: Intracellular calcium current disorder and disease phenotype in OBSCN mutant iPSC-based cardiomyocytes in arrhythmogenic right ventricular cardiomyopathy
Source: Theranostics. 2020 Sep 14;10(24):11215–29. doi: 10.7150/thno.45172 (PMC7532677; doi:10.7150/thno.45172)

Table S2-- Comparison of task force criteria and patients' conditions

**2010 Task force criteria** [1]	**ARVC patient**
I. Global or regional dysfunction and structural alterations<br>**Major**<br>By 2D echo:<br>• Regional RV akinesia, dyskinesia, or aneurysm and one of the following (end diastole):<br>— PLAX RVOT ≥32 mm (corrected for body size [PLAX/BSA] ≥19 mm/m2)<br>— PSAX RVOT ≥36 mm (corrected for body size [PSAX/BSA] ≥21 mm/m2)<br>— or fractional area change ≤33%<br><br>By MRI:<br>• Regional RV akinesia or dyskinesia or dyssynchronous RV contraction and one of the following:<br>— Ratio of RV end-diastolic volume to BSA ≥110 mL/m2 (male) or ≥100 mL/m2 (female)<br>— or RV ejection fraction ≤40%<br><br>By RV angiography:<br>• Regional RV akinesia, dyskinesia, or aneurysm<br><br>**Minor**<br>By 2D echo:<br>• Regional RV akinesia or dyskinesia and one of the following (end diastole):<br>— PLAX RVOT ≥29 to <32 mm (corrected for body size [PLAX/BSA] ≥16 to <19 mm/m2)<br>— PSAX RVOT ≥32 to <36 mm (corrected for body size [PSAX/BSA] ≥18 to <21 mm/m2)<br>— or fractional area change >33% to ≤40%<br><br>By MRI:	**Major**<br>The patient had regional RV dyskinesia and PLAX/BSA RVOT ≥21 mm/m^2 by 2D echo.

• Regional RV akinesia or dyskinesia or dyssynchronous RV contraction and one of the following:<br>— Ratio of RV end-diastolic volume to BSA ≥100 to <110 mL/m2 (male) or ≥90 to <100 mL/m2 (female)<br>— or RV ejection fraction >40% to ≤45%<br><br>PLAX indicates parasternal long-axis view; RVOT, RV outflow tract; BSA, body surface area; PSAX, parasternal short-axis view; aVF, augmented voltage unipolar left foot lead; and aVL, augmented voltage unipolar left arm lead.	
II. Tissue characterization of wall<br>**Major**<br>• Fibrofatty replacement of myocardium on endomyocardial biopsy<br>• Residual myocytes <60% by morphometric analysis (or <50% if estimated), with the fibrous replacement of the RV free wall myocardium in ≥1 sample, with or without fatty replacement of tissue on endomyocardial biopsy<br>**Minor**<br>• Residual myocytes 60% to 75% by morphometric analysis (or 50% to 65% if estimated), with the fibrous replacement of the RV free wall myocardium in ≥1 sample, with or without fatty replacement of tissue on endomyocardial biopsy	**Undetected**
III. Repolarization abnormalities<br>**Major**<br>• Inverted T waves in the right precordial leads (V_1, V_2, and V_3) or beyond in individuals >14 years of age (in the absence of complete right bundle-branch block QRS ≥120 ms)<br>**Minor**<br>• Inverted T waves in leads V_1 and V_2 in individuals >14 years of age (in the absence of complete right bundle-branch block) or in V_4, V_5, or V_6<br>• Inverted T waves in leads V1, V2, V3, and V4 in individuals >14 years of age in the presence of complete right bundle-branch block	**No match**<br><br>Inverted T waves in leads V1, V2, V3, in individuals >14 years of age in the presence of complete right bundle-branch block
IV. Depolarization/conduction abnormalities<br>**Major**	**Major**<br>Epsilon wave (reproducible low-amplitude signals

• Epsilon wave (reproducible low-amplitude signals between the end of QRS complex to the onset of the T wave) in the right precordial leads (V_1 to V_3)<br>**Minor**<br>• Late potentials by SAECG in ≥1 of 3 parameters in the absence of a QRS duration of ≥110 ms on the standard ECG<br>• Filtered QRS duration (fQRS) ≥114 ms<br>• Duration of terminal QRS <40 μV (low-amplitude signal duration) ≥38 ms<br>• Root-mean-square voltage of terminal 40 ms ≤20 μV<br>• Terminal activation duration of QRS ≥55 ms measured from the nadir of the S wave to the end of the QRS, including R′, in V_1, V_2, or V_3, in the absence of complete right bundle-branch block	between end of QRS complex to onset of the T wave) in the right precordial leads (V_1 to V_3)
V. Arrhythmias<br>**Major**<br>• Nonsustained or sustained ventricular tachycardia of left bundle-branch morphology with a superior axis (negative or indeterminate QRS in leads II, III, and aVF and positive in lead aVL)<br><br>**Minor**<br>• Nonsustained or sustained ventricular tachycardia of RV outflow configuration, left bundle-branch block morphology with the inferior axis (positive QRS in leads II, III, and aVF and negative in lead aVL) or of an unknown axis<br>• > 500 ventricular extrasystoles per 24 hours (Holter)	**Minor**<br>Sustained ventricular tachycardia of unknown axis
VI. Family history<br>**Major**<br>• ARVC/D confirmed in a first-degree relative who met current Task Force criteria<br>• ARVC/D confirmed pathologically at autopsy or surgery in a first-degree relative<br>• Identification of a pathogenic mutation† categorized as associated or probably associated with ARVC/D in the patient under evaluation<br><br>**Minor**	**No match**

• History of ARVC/D in a first-degree relative in whom it was not possible or practical to determine whether the family member met current Task Force criteria<br>• Premature sudden death (<35 years of age) due to suspected ARVC/D in a first-degree relative<br>• ARVC/D confirmed pathologically or by current Task Force Criteria in a second-degree relative	
**Definite diagnosis:** 2 major, or 1 major and 2 minor, or 4 minor criteria from different categories; borderline diagnosis: 1 major and 1 minor, or 3 minor criteria from different categories; possible diagnosis: 1 major, or 2 minor criteria from different categories.	**2 major and 1 minor criteria**

References

1. Marcus FI, McKenna WJ, Sherrill D, Basso C, Bauce B, Bluemke DA, et al. Diagnosis of arrhythmogenic right ventricular cardiomyopathy/dysplasia: proposed modification of the Task Force Criteria. Eur Heart J. 2010; 31: 806-14.

Table S3. Primers for real-time PCR

Gene	Forward (5' to 3')	Reverse (5' to 3')
GAPDH	GGTGAAGGTCGGAGTCAACGGATTTGGTCG	GGATCTCGCTCCTGGAAGATGGTGATGGG
OBSCN	GCTGAGCTCCGTGTGGACT	CAGTGGTGGACTCTGTTCCCT
ANK1.5	CCAGATGAATGGTTACTCCTCAC	CAAGGGGATGGCGTCTAGG
POU5F1	CAGTGCCCGAAACCCACAC	GGAGACCCAGCAGCCTCAAA
NANOG	CAGAAGGCCTCAGCACCTAC	ATTGTTCCAGGTCTGGTTGC
α-actinin	TCCATCGGAGCCGAAGAAATC	GTGTCGGTGGATCAAAGCACA
plakoglobin	TCAGCAGCAAGGGCATCAT	TGGGTGTAAGTGGTGGTTTTCTT
plakophilin2	GCAAATGGTTTGCTCGATTT	GGCTGGTAATCTGCAATGGT
N-cadherin	CCTGCTTCAGGCGTCTGTAG	CTGCCTTTGTAGGTGGCCAC
connexin43	GGAATGCAAGAGAGGTTGAAAG	GGCATTTGGAGAAACTGGTAGA
desmoplakin	CAGTGGTGTCAGCGATGATGT	TGACGCTGGATATGGTGGAA
C/EBPα	CACGAAGCACGATCAGTCCAT	CGCACATTCACATTGCACAAG
FABP4	TGGTGGTGGAATGCGTCAT	GGTCAACGTCCCTTGGCTTA
PPARγ	AGGCCATTTTCTCAAACGAG	CCATTACGGAGAGATCCACG

Figure S1-Recording of implantable cardioverter defibrillator

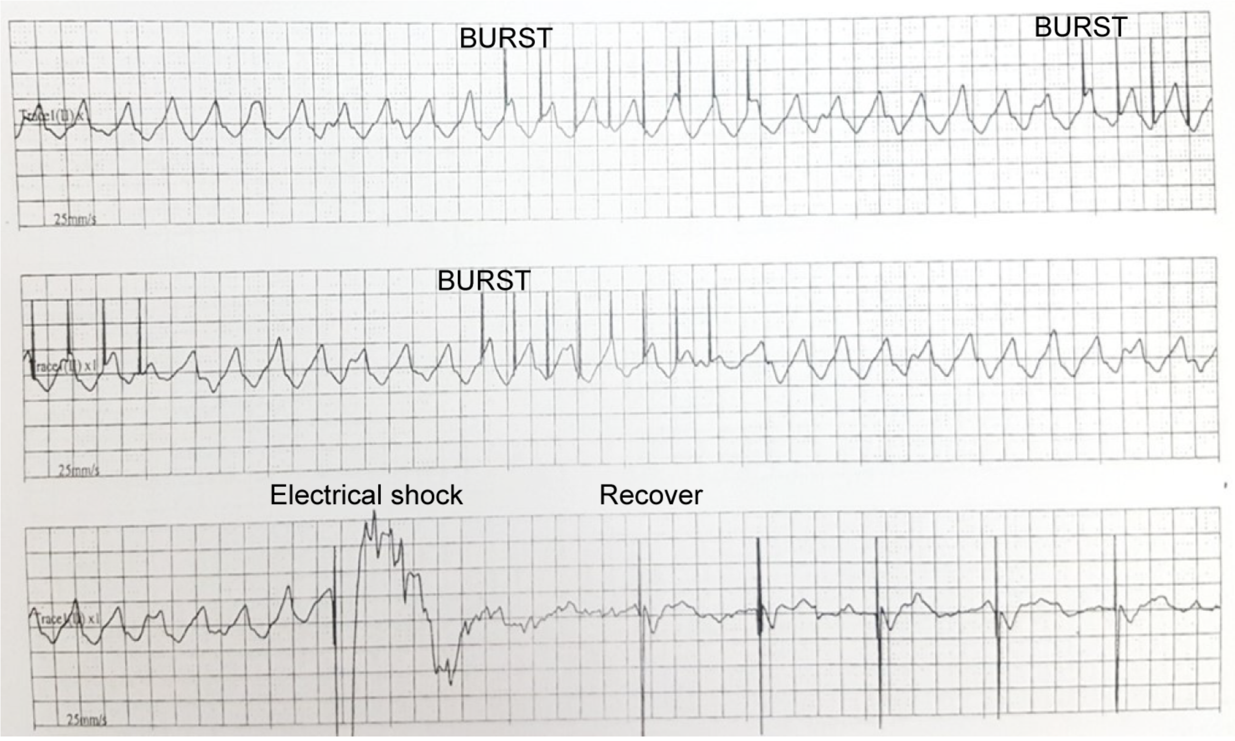

Figure S2-- Alignment of sequences of the *OBSCN* gene from human, tupaia, vulpes, microtus, nothoprocta, vulpes and zonotrichia.

∗The asterisk represents conservative sequences. The red box is the location of the target gene mutation in the study.

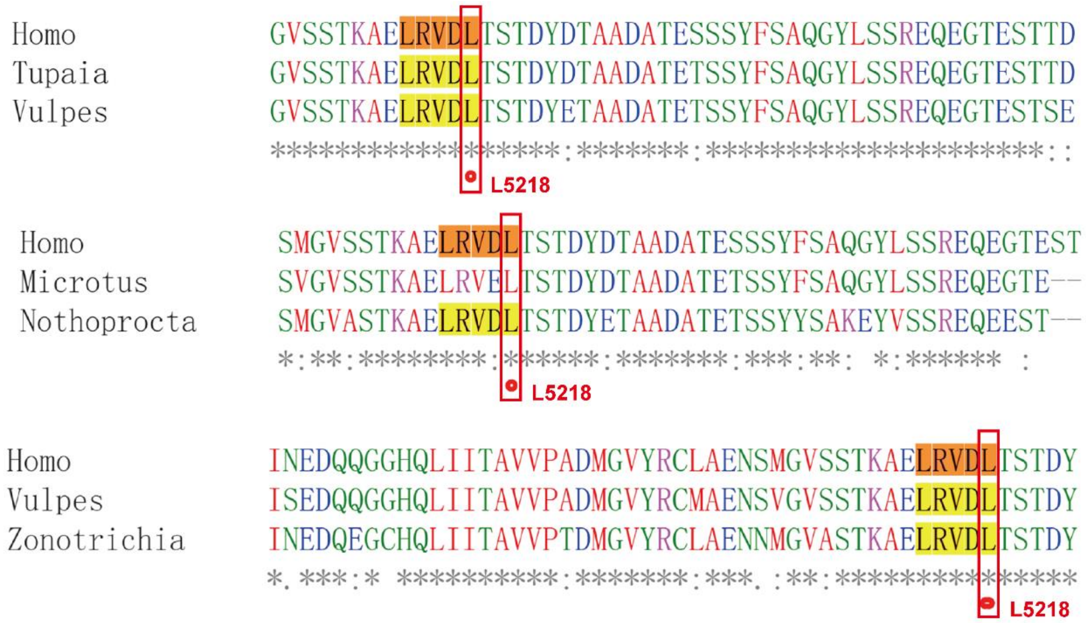

Supplement: Supplementary file 1 — Supplementary data including figures and tables. [file thnov10p11215s1.pdf]
